# Tuberculosis progression rates in U.S. Immigrants following screening with interferon-gamma release assays

**DOI:** 10.1186/s12889-016-3519-6

**Published:** 2016-08-25

**Authors:** Robert J. Blount, Minh-Chi Tran, Charles K. Everett, Adithya Cattamanchi, John Z. Metcalfe, Denise Connor, Cecily R. Miller, Jennifer Grinsdale, Julie Higashi, Payam Nahid

**Affiliations:** 1 Division of Pulmonary and Critical Care Medicine, San Francisco General Hospital, University of California San Francisco, San Francisco, CA USA; 2 Division of Pediatric Pulmonary Medicine, University of California, San Francisco, CA USA; 3 Department of Internal Medicine, University of California, Davis, Sacramento, CA USA; 4 Department of Medicine, University of California, San Francisco, CA USA; 5 San Francisco Department of Public Health, Population Health Division, Office of Equity and Quality Improvement, San Francisco, CA USA; 6 San Francisco Department of Public Health, Population Health Division, Disease Prevention and Control Branch, San Francisco, CA USA

**Keywords:** Interferon-gamma release assay (IGRA), Tuberculosis (TB), Latent tuberculosis infection (LTBI), Active tuberculosis disease, Foreign-born, Immigrant, Incidence rate, Chest x-ray (CXR), Preventive chemotherapy

## Abstract

**Background:**

Interferon-gamma release assays may be used as an alternative to the tuberculin skin test for detection of *M. tuberculosis* infection. However, the risk of active tuberculosis disease following screening using interferon-gamma release assays in immigrants is not well defined. To address these uncertainties, we determined the incidence rates of active tuberculosis disease in a cohort of high-risk immigrants with Class B TB screened with interferon-gamma release assays (IGRAs) upon arrival in the United States.

**Methods:**

Using a retrospective cohort design, we enrolled recent U.S. immigrants with Class B TB who were screened with an IGRA (QuantiFERON ® Gold or Gold In-Tube Assay) at the San Francisco Department of Public Health Tuberculosis Control Clinic from January 2005 through December 2010. We reviewed records from the Tuberculosis Control Patient Management Database and from the California Department of Public Health Tuberculosis Case Registry to determine incident cases of active tuberculosis disease through February 2015.

**Results:**

Of 1233 eligible immigrants with IGRA screening at baseline, 81 (6.6 %) were diagnosed with active tuberculosis disease as a result of their initial evaluation. Of the remaining 1152 participants without active tuberculosis disease at baseline, 513 tested IGRA-positive and 639 tested IGRA-negative. Seven participants developed incident active tuberculosis disease over 7730 person-years of follow-up, for an incidence rate of 91 per 100,000 person-years (95 % CI 43–190). Five IGRA-positive and two IGRA-negative participants developed active tuberculosis disease (incidence rates 139 per 100,000 person-years (95 % CI 58–335) and 48 per 100,000 person-years (95 % CI 12–193), respectively) for an unadjusted incidence rate ratio of 2.9 (95 % CI 0.5–30, *p* = 0.21). IGRA test results had a negative predictive value of 99.7 % but a positive predictive value of only 0.97 %.

**Conclusions:**

Among high-risk immigrants without active tuberculosis disease at the time of entry into the United States, risk of progression to active tuberculosis disease was higher in IGRA-positive participants compared with IGRA-negative participants. However, these findings did not reach statistical significance, and a positive IGRA at enrollment had a poor predictive value for progressing to active tuberculosis disease. Additional research is needed to identify biomarkers and develop clinical algorithms that can better predict progression to active tuberculosis disease among U.S. immigrants.

## Background

The number of active tuberculosis (TB) cases in the United States (U.S.) has declined each year since 1993, yet the proportion of cases in foreign-born persons is steadily rising [1]. In 2014, foreign-born persons accounted for 66.5 % of total cases in the U.S. and had a 13.4 times higher incidence rate of active TB disease compared with U.S.-born persons [2]. Additionally, up to 84 % of active TB in foreign-born persons can be attributed to reactivation of an infection acquired prior to arrival in the U.S. [3–5]. Accurately identifying and treating foreign-born persons with latent tuberculosis infection (LTBI) who are at high risk for progression to active TB disease should improve overall TB control in the U.S. [6–9].

Interferon-gamma release assays (IGRAs) measure interferon-gamma secretion following stimulation of T-lymphocytes with *Mycobacterium tuberculosis* antigens [10], and may be used as an alternative to the tuberculin skin test (TST) [1, 10]. Several studies conducted in close contacts of active TB index cases have reported a higher active TB incidence in IGRA-positive persons compared with IGRA-negative persons [11–17]. However, few studies have addressed this question in the evaluation of high-risk newly arriving immigrants [18, 19].

Since 2005, the San Francisco Department of Public Health (SFDPH) TB Control Clinic has incorporated IGRAs into routine clinical practice according to Centers for Disease Control and Prevention (CDC) guidelines [20]. We analyzed data from the SFDPH TB Control Patient Management Database and the California TB Case Registry to provide program-relevant guidance on the management of foreign-born patients screened with IGRAs. Our primary objective was to determine the risk of progression to active TB disease among immigrants screened for LTBI with an IGRA within 1 year of arrival into the United States.

## Methods

### Study population

The SFDPH TB Control Clinic evaluates and treats patients with suspected active TB, prior TB, and suspected LTBI in accordance with American Thoracic Society (ATS), CDC, and Infectious Diseases Society of America (IDSA) guidelines [21, 22]. Among these patients are immigrants, refugees, and asylum-seekers. As part of the immigrant medical evaluation for TB, patients may have had a TST or IGRA, a chest radiograph CXR, and/or sputum collected for smear and/or culture. Recommendations for immigration evaluation have changed during the study period [9, 23–25]. Currently, visa applicants outside the U.S. are screened with CXR followed by sputum examination if the CXR is suspicious, whereas for those residing in the U.S. at the time of application or are <15 years old, screening involves a TST or IGRA and if either is positive, a CXR is obtained. From this evaluation visa applicants are assigned a TB classification status by the U.S. Citizenship and Immigration Services (Table 1). All persons with Class B TB are then referred to their local jurisdiction TB control program upon U.S. arrival for further clinical evaluation [25–27]. The Class B TB classification scheme has been modified during the study period and currently includes those with possible smear and culture negative pulmonary TB (B1-Pulmonary), those with a history of successfully treated pulmonary TB (B1-Pulmonary), those with extrapulmonary TB (B1-Extrapulmonary), those with a positive TST or IGRA but no evidence of active TB (B2), and those in close contact with a known case of smear or culture positive pulmonary TB but with a negative TST or IGRA (B3). Prior to 2008, Class B immigrants also included those who had radiographic evidence of old/healed TB. All children and adults with B1-Pulmonary, B2, and B3 TB were offered IGRA screening at the SFDPH TB Control Clinic during the enrollment period of January 1, 2005 through December 31, 2010, and those who received IGRA testing were considered for inclusion in our study. The IGRA assays (QuantiFERON-TB Gold® from January 1, 2005 through December 31, 2007 and QuantiFERON®-TB Gold In-Tube from January 1, 2008 through December 31, 2010, Cellestis Limited, Carnegie, Australia) were performed at the SFDPH laboratory according to manufacturers’ instructions and interpreted using FDA approved criteria [20]. In addition to IGRA testing, tuberculin skin tests (Tubersol, 5TU from Sanofi Pasteur Limited, Toronto, Canada) were offered at the discretion of the attending physician, performed according to manufacturer guidelines, and interpreted by clinic staff according to CDC guidelines [28]. Patients diagnosed with LTBI at the time of enrollment were offered preventive chemotherapy if they had not yet received adequate therapy overseas. Patients typically self-administered their medication, coming in to the TB clinic for monthly refills. Adherence was monitored at monthly clinic visits, and completion of therapy documented once the required course of preventive chemotherapy had be taken per patient report. We excluded persons who were evaluated more than 1 year following their immigration date or had persistently indeterminate IGRA results despite repeat testing. Patients who were diagnosed with or treated for active TB disease for > 30 days as a direct result of their initial evaluation at the TB Control Clinic were counted as prevalent cases and excluded from the final analysis. Clinical, radiologic, and laboratory data were written on standardized forms in the medical record and then entered into the SFDPH TB Control Patient Management Database. Study treatment status was reflective of any documented TB treatment received either before or after U.S. entry and throughout the followup period up until a diagnosis of active TB or the end of the study, whichever came first.

**Table 1:** Current tuberculosis classification for U.S. immigration applicants

TB classification Status	Description
No TB classification	No evidence of TB infection or disease
A	Smear or culture^a^ positive pulmonary TB. Typically requires treatment prior to U.S. entry.
B1-Pulmonary	1. Suspected active pulmonary TB based on clinical findings but with negative sputum smears and cultures^a^
	2. History of pulmonary TB, treatment complete
B1-Extrapulmonary	Extrapulmonary TB
B2	TST ≥ 10 mm or positive IGRA but otherwise negative TB evaluation (LTBI)
B3	Recent close contact with a known active TB case and TST/IGRA negative

*Abbreviations*: *TB* tuberculosis, *LTBI* latent tuberculosis infection, *TST* tuberculin skin test

^a^Cultures were gradually introduced into the overseas screening process from 2007–2014

### Study outcome

Follow-up time began on the day of IGRA testing at the initial SFDPH TB Control Clinic visit and was censored on the day of active TB diagnosis (event), death, or the end of the study (February 2015). We identified active TB cases during follow-up using the SFDPH TB Control Patient Management Database and the California Department of Public Health TB Case Registry. All incident active TB cases were microbiologically or pathologically confirmed. We performed a death registry match with the California Department of Public Health Vital Statistics Office to determine mortality through December 2009. We did not update this match in 2015 due to low yield, finding only four deaths in the initial assessment, none of which were known to be attributable to TB. We classified active TB cases by adapting an existing classification scheme [29]: 1) “likely LTBI reactivation” if the IGRA was positive at enrollment but the immigration chest X-ray (CXR) was normal; 2) “likely reactivation of prior TB” if the immigration CXR was abnormal but the participant was not diagnosed with active TB disease as a result of the enrollment evaluation; 3) “likely imported active TB” if the immigration CXR was abnormal and the participant was diagnosed with active TB disease as a result of the enrollment evaluation; and 4) “likely TB infection and disease acquired after U.S. immigration” if the participant was IGRA-negative with a normal CXR at the time of enrollment. CXRs were performed overseas near the time of immigration and were repeated upon arrival in the U.S. CXRs were interpreted by physicians at the SFDPH TB Control Clinic during the baseline evaluation. The CXR performed in the U.S. closest to baseline evaluation was used as the CXR interpretation for the study. The IGRA and TST results reported in this study were from the post-arrival baseline evaluation.

### Statistical analysis

We used Pearson’s chi-squared test for comparisons of proportions and Student’s *T*-test for comparisons of means. We calculated unadjusted incidence and incidence rate ratios (IRR) with 95 % confidence intervals per 100,000 person-years of follow-up, with statistical significance defined as an IRR *p* value < 0.05. We estimated the cumulative incidence of active TB disease using Kaplan-Meier curves. We determined IGRA test performance by calculating sensitivity, specificity, positive and negative predictive value, and positive and negative likelihood ratios. We performed statistical analyses using Stata/SE 13.1 (College Station, Texas, USA).

## Results

### Baseline demographic and clinical characteristics

**Fig. 1:**
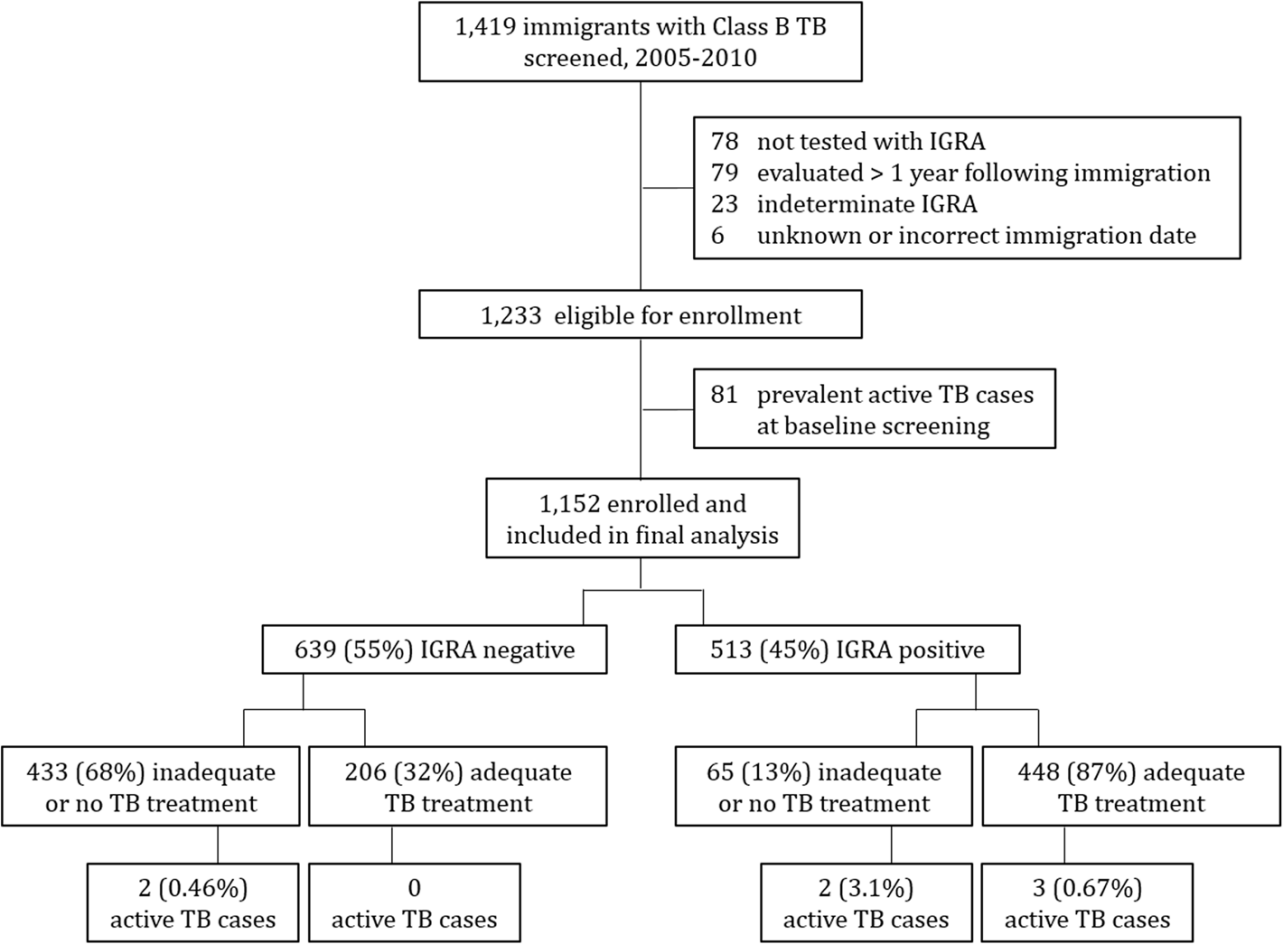
Enrollment flow chart

During the enrollment period January 1, 2005 through December 31, 2010 we screened 1,419 Class B TB immigrants for LTBI. We excluded 78 (5.5 %) who did not have IGRA testing completed, 79 (5.6 %) who were evaluated > 1 year following immigration, 23 (1.6 %) who had indeterminate IGRA results despite repeat testing, and 6 (0.4 %) who had an unknown or incorrect immigration date (Fig. 1). From the remaining 1,233 eligible participants, 81 (6.6 %) were found to have active TB disease during enrollment screening. All but one prevalent case, and all of the 42 microbiologically confirmed prevalent cases, had abnormal immigration CXRs at the time of U.S. entry. IGRA was positive in 54 (67 %) of total prevalent cases and in 34 (81 %) of microbiologically confirmed prevalent cases. All prevalent cases were then excluded, while the remaining 1,152 subjects were included in the final analysis. The average time from immigration to IGRA testing was 32 days (SD 42). We examined baseline demographic and clinical characteristics by IGRA test results: 639 (55 %) were IGRA-negative and 513 (45 %) were IGRA-positive (Table 2). Median age was 54 (IQR 39–64) years and IGRA-positive participants were significantly older than those with IGRA-negative test results at enrollment (*p* < 0.0001). Of the 973 participants over the age of 21, 494 (51 %) were IGRA-positive at enrollment, whereas of the 179 participants under the age of 21, only 19 (11 %) were IGRA-positive. The majority of participants were male (*n* = 645, 56 %), and 1066 (93 %) emigrated from one of three countries: China, the Philippines, or Vietnam. Baseline TB risk factors were assessed: only 0.25 % of participants had known HIV-infection, 5.4 % had diabetes, while 30 % reported current or former tobacco smoking. IGRA-positive participants were more likely to be smokers than IGRA-negative participants (*p* < 0.0001). Nearly half (49 %) of participants immigrated with a B1-Pulmonary classification (TB suspect or prior TB), while 51 % received an overseas classification of B2 (LTBI). Among IGRA-positive participants, 87 % had received either overseas treatment for TB infection or disease prior to immigration (*n* = 188) or LTBI treatment upon immigration in the U.S. (*n* = 260). Tuberculin skin testing was performed in only 298 participants with a similar percent positive TST in IGRA-negative participants (76 %) as in IGRA-positive participants (79 %) (*p* = 0.5). TST test results correlated poorly with IGRA test results (*r* = 0.037). CXR results were available for nearly all participants (*n* = 1150) and found to be abnormal in 881 (77 %). IGRA-positive participants were more likely to have an abnormal CXR compared with IGRA-negative participants (90 vs 66 %, *p* < 0.0001).

**Table 2:** Baseline demographic and clinical characteristics by IGRA status

Characteristic	All subjects	IGRA-negative	IGRA-positive	*p* value
	n (%)^a^	n (%)	n (%)	
All	1152 (100)	639 (55)	513 (45)	
Median age, years (IQR)	54 (39–64)	52 (21–64)	55 (45–65)	
Mean age, years (SD)	49 (20)	45 (23)	53 (15)	<0.0001
Age by category, years, n (%)	*n* = 1152	*n* = 639	*n* = 513	<0.0001
0–4	18 (1.6)	17 (2.7)	1 (0.2)	
5–12	87 (7.6)	81 (13)	6 (1.2)	
13–20	74 (6.4)	62 (9.7)	12 (2.3)	
≥ 21	973 (84)	479 (75)	494 (96)	
Gender	*n* = 1152	*n* = 639	*n* = 513	0.141
Male	645 (56)	345 (54)	300 (58)	
Female	507 (44)	294 (46)	213 (42)	
Country of origin	*n* = 1150	*n* = 638	*n* = 512	0.001
China	586 (51)	302 (47)	284 (55)	
Philippines	386 (34)	245 (38)	141 (28)	
Vietnam	94 (8.2)	42 (6.6)	52 (10)	
Burma	18 (1.6)	11 (1.7)	7 (1.4)	
Other	66 (5.7)	38 (6.0)	28 (5.4)	
HIV status	*n* = 799	*n* = 404	*n* = 395	<0.0001
HIV-	797 (99.8)	402 (99.5)	395 (100)	
HIV+	2 (0.25)	2 (0.5)	0	
Diabetes	*n* = 1152	*n* = 639	*n* = 513	0.09
No	1090 (95)	611 (96)	479 (93)	
Yes	62 (5.4)	28 (4.4)	34 (6.6)	
Smoking status	*n* = 1152	*n* = 639	*n* = 513	<0.0001
Never	803 (70)	476 (74)	327 (64)	
Current or Former	349 (30)	163 (26)	186 (36)	
TB Classification at immigration	*n* = 1152	*n* = 639	*n* = 513	0.0001
B1-Pulmonary (TB suspect or prior TB)	561 (49)	276 (43)	285 (56)	
B2 (LTBI)	588 (51)	361 (56)	227 (44)	
B3 (Contact)	3 (0.26)	2 (0.31)	1 (0.19)	
BCG	*n* = 490	*n* = 309	*n* = 181	0.004
No evidence of BCG vaccination	114 (23)	59 (19)	55 (30)	
Evidence of BCG vaccination	376 (77)	250 (81)	126 (70)	
Treatment status^b^	*n* = 1152	*n* = 639	*n* = 513	<0.0001
Inadequate or no TB treatment	498 (43)	433 (68)	65 (13)	
Overseas TB treatment prior to immigration	324 (28)	136 (21)	188 (37)	
LTBI treatment in U.S. after immigration	330 (29)	70 (11)	260 (51)	
TST Result	*n* = 298	*n* = 230	*n* = 68	0.5
Negative	70 (23)	56 (24)	14 (21)	
Positive	228 (77)	174 (76)	54 (79)	
Immigration CXR Result	*n* = 1150	*n* = 637	*n* = 513	<0.0001
Normal	269 (23)	216 (34)	53 (10)	
Abnormal	881(77)	421 (66)	460 (90)	
Prevalent TB cases at enrollment				
Total	81/1233 (6.6)	27/666 (4.1)	54/567 (9.5)	0.0001
Microbiologically confirmed	42/1233 (3.4)	8/666 (1.2)	34/567 (6.0)	<0.0001

*Abbreviations*: *IGRA* interferon-gamma release assay, *IQR* interquartile range, *HIV* human immunodeficiency virus, *TST* tuberculin skin test, *TB* tuberculosis, *CXR* chest x-ray

^a^Table numbers represent n (%) unless otherwise indicated

^b^Treatment status reflects any TB treatment received up until the time of diagnosis with active TB disease: either overseas treatment for TB infection or disease prior to U.S. entry, or LTBI treatment received in the U.S. after immigration

### Incident active TB cases

Seven participants (0.6 %) developed incident active TB disease over the 10 year follow-up period (Table 3). The majority of cases (57 %) were male, all were adults between the ages of 39 and 60 (median 52) at the time of enrollment, and all but one case had emigrated from China. Five (71 %) were IGRA-positive while two were IGRA-negative at enrollment. Case #5 was likely LTBI reactivation as the CXR performed 1 week after arrival in the U.S. was normal and the IGRA at that time was positive. This participant was diagnosed with active TB disease latest at 6.3 years after enrollment. The other six cases (86 %) were likely reactivation of prior TB, having immigration CXR findings in the upper lobes consistent with prior TB. These participants were diagnosed with active TB disease a median of 4.5 years after enrollment (IQR 3.7–4.8). None of the cases had known HIV, diabetes, or tobacco smoking risk factors. Three of the five cases that were IGRA-positive at enrollment completed LTBI treatment after enrollment. Diagnosis was confirmed with sputum culture for six cases and by lung biopsy pathologic features (caseating granulomatous inflammation) for one case.

**Table 3:** Incident active TB cases

Case	IGRA	Ag-Nil, Mitogen (IU/ml)	CXR findings	HIV	Diabetes	Smoker	TB Class	Treatment status^a^	TB confirmation
1	Neg	0.38–0.11, 20	Pleural thickening, RUL	Neg	No	No	B1	None	Lung biopsy pathology
2	Pos	0.93–0.12, 2.0	Fibronodular changes, BUL & LLL	Unk	No	No	B2	None	sputum culture
3	Neg	0.29–0.11, 25	Fibrotic changes, LUL	Neg	No	No	B2	Incomplete tx prior to immigration^b^	sputum culture
4	Pos	2.4–0.12, 25	Fibronodular changes, RUL	Neg	No	No	B1	LTBI tx at enrollment	sputum culture
5	Pos	9.7–0.70, 24	Normal	Neg	No	No	B1	LTBI tx at enrollment	sputum culture
6	Pos	3.1–0.12, 8.9	Nodular infiltrate, LUL	Neg	No	No	B1	LTBI tx at enrollment	sputum culture
7	Pos	1.1–0.16, 6.9	Fibrotic changes, LUL; Effusion, L	Neg	No	No	B1	Incomplete LTBI tx at enrollment^c^	sputum culture

*Abbreviations*: *TB* tuberculosis, *IGRA* interferon-gamma release assay, *Ag-Nil* interferon-gamma response with antigen minus response without antigen, *Mitogen* interferon-gamma response to mitogen, *HIV* human immunodeficiency virus, *CXR* chest x-ray, *Pos* positive, *Neg* negative, *Unk* unknown, *RUL* right upper lobe, *BUL* bilateral upper lobes, *LLL* left lower lobe, *LUL* left upper lobe, *L* left, *LTBI* latent tuberculosis infection, *tx* treatment

^a^Treatment status reflects any TB treatment received up until the time of diagnosis with active TB disease: either overseas treatment for TB infection or disease prior to U.S. entry, or LTBI treatment received in the U.S. after immigration

^b^Defaulted after 3 months of active TB treatment

^c^Received a 1 month prescription for isoniazid but was lost to follow-up. Unclear if patient took any of the medication

### Incidence rates for active TB disease during follow-up

**Fig. 2:**
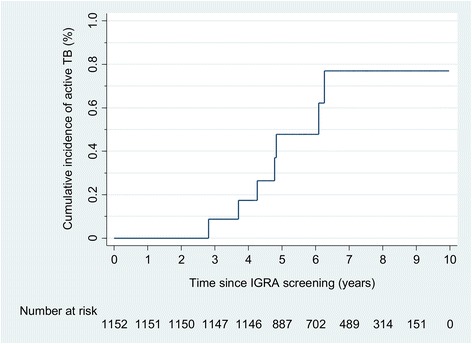
Cumulative incidence of active tuberculosis (TB) disease among high-risk U.S. immigrants, 2005–2015

The seven incident active TB cases occurred over 7730 person-years of cumulative follow-up, for a median follow-up of 6.7 years (IQR 5.1–8.2) and an overall incidence rate of 91 per 100,000 person-years (95 % CI 43–190) (Table 4). Incidence rates for active TB disease diagnosed during follow-up were higher in IGRA-positive participants (139 per 100,000 person-years, 95 % CI 58–335) compared with those who were IGRA-negative at enrollment (48 per 100,000 person-years, 95 % CI 12–193), for an unadjusted incidence rate ratio (IRR) of 2.9 (95 % CI 0.5–30, *p* = 0.21). Additionally, active TB incidence rates were higher in those with inadequate or no prior TB treatment (127 per 100,000 person-years, 95 % CI 48–339) compared to those with prior TB treatment (65 per 100,000 person-years 95 %, CI 21–203), for an IRR of 1.9 (95 % CI 0.33–13, *p* = 0.41). These differences, however, did not reach statistical significance. The risk for progression to active TB disease was highest among IGRA-positive participants who had received inadequate or no TB treatment before and after U.S. arrival (incidence rate 443 per 100,000 person-years, 95 % CI 111–1775), while risk was lower in IGRA-positive participants who received TB treatment (incidence rate 96 per 100,000 person-years, 95 % CI 31–296), for an IRR of 4.6 (95 % CI 0.39–41, *p* = 0.14). The risk was lowest among IGRA-negative participants who had received prior treatment for TB (incidence rate of 0) and IGRA-negative participants who had a normal immigration CXR (incidence rate of 0). For the entire cohort we found a 5 year cumulative incidence of 0.48 % and a 10 year cumulative incidence of 0.77 % (Fig. 2).

**Table 4:** Active TB incidence rates for treatment status, CXR findings, and overseas TB classification by IGRA result

Treatment status,^a^ CXR, and TB class by IGRA result	n (% of all participants)	Incident active TB cases, n (% cases in sub-group)	Total follow-up (person-years)	Incidence rate per 100,000 person-years (95 % CI)
All participants	1152 (100)	7 (0.60)	7730	91 (43–190)
IGRA-	639 (55)	2 (0.31)	4139	48 (12–193)
IGRA+	513 (45)	5 (0.97)	3591	139 (58–335)
Inadequate or no TB treatment	498 (43)	4 (0.80)	3148	127 (48–339)
IGRA-	433 (38)	2 (0.46)	2698	74 (19–296)
IGRA+	65 (6.0)	2 (3.1)	451	443 (111–1775)
Adequate TB treatment	654 (57)	3 (0.46)	4581	65 (21–203)
IGRA-	206 (18)	0	1441	0
IGRA+	448 (39)	3 (0.67)	3140	96 (31–296)
Normal CXR^b^	269 (23)	1 (0.37)	1553	64 (9.1–457)
IGRA-	216 (19)	0	1206	0
IGRA+	53 (4.6)	1 (1.9)	347	288 (41–2047)
Abnormal CXR	881 (77)	6 (0.68)	6158	97 (44–217)
IGRA-	421 (37)	2 (0.48)	2914	69 (17–274)
IGRA+	460 (40)	4 (0.87)	3244	123 (46–329)
B1 TB, Pulmonary	561 (49)	5 (0.89)	3630	138 (57–331)
IGRA-	276 (24)	1 (0.36)	1759	57 (8.0–404)
IGRA+	285 (25)	4 (1.4)	1870	214 (80–570)
B2 (LTBI)	588 (51)	2 (0.34)	4084	49 (12–196)
IGRA-	361 (31)	1 (0.28)	2370	42 (5.9–300)
IGRA+	227 (20)	1 (0.44)	1714	58 (8.2–414)
B3 (Contacts)	3 (0.26)	0	16	0

*Abbreviations*: *TB* tuberculosis, *IGRA* interferon-gamma release assay

^a^Treatment status reflects any TB treatment received up until the time of diagnosis with active TB: either overseas treatment for TB infection or disease prior to U.S. entry, or LTBI treatment received in the U.S. after immigration

^b^ *n* = 1150 (2 participants missing CXRs)

### IGRA test characteristics

The IGRA test had a sensitivity of 71 % (95 % CI 29-96 %) for predicting progression to active TB disease, a specificity of 56 % (95 % CI 53–59 %), a positive predictive value of only 0.97 % (95 % CI 0.32–2.3 %), and a negative predictive value of 99.7 % (95 % CI 98.9–100 %). The positive likelihood ratio was 1.6 (95 % CI 1.0–2.6) and the negative likelihood ratio was 0.51 (95 % CI 0.16–1.7). Among IGRA-positive participants with nil values ≤ 0.7 IU/ml, there was not a statistically significant difference in TB response values at baseline among those that developed active TB (median 2.3 IU/ml, IQR 0.93–3.0) compared with those who did not develop active TB (1.6, 0.71–4.0) (*p* = 0.6).

## Discussion

We evaluated the rates of progression to active TB disease among a cohort of high-risk immigrants screened for LTBI with IGRAs at the San Francisco Department of Public Health TB Control Clinic in a large study of 1152 participants with 7730 person-years of follow-up time. We found a high prevalence of active TB disease at baseline evaluation and a high incidence of active TB disease over the median follow-up time of 6.7 years. The incidence rates of active TB disease were higher in IGRA-positive participants compared with those who were IGRA-negative at enrollment. However, these findings were not statistically significant and a positive IGRA at enrollment had a poor predictive value for progression to active TB disease.

Our findings are largely consistent with a prior meta-analysis and a recent prospective cohort study from Europe, both of which found that IGRAs had poor predictive value for active TB disease in one or more high-risk groups (case contacts, health care workers, immigrants, people living with HIV, and patients with silicosis) [11, 12]. In our cohort, the risk of developing active TB disease was high regardless of IGRA results. The overall incidence rate of 91 per 100,000 person-years and even the incidence rate for IGRA-negative participants (48 per 100,000 person years) were considerably higher than the incidence for U.S.-born persons, which decreased from 2.5 cases per 100,000 persons in 2005 to 1.1 cases per 100,000 persons in 2014 [2, 30].

In our cohort there was a heightened risk of developing active TB among Class B immigrants with CXR evidence of prior TB. This elevated risk was sustained over several years, with incident active TB disease occurring from 2.8 to 6.3 years after enrollment and 10 year cumulative incidence nearly double the 5 year cumulative incidence. The sustained high incidence is consistent with other studies of immigrants from TB-endemic countries [1, 29, 31], suggesting that foreign-born persons from TB-endemic countries should be screened for LTBI regardless of how recently they immigrated to the U.S., and that a high level of suspicion for active TB disease should be maintained in this group even when IGRA results are negative.

Our findings confirm the importance of radiographic evidence of prior TB as a key risk factor for developing TB, particularly when overseas treatment is uncertain or absent [29]. Six of the seven immigrants who developed active TB disease had upper lobe abnormalities on their immigration CXR. None had overseas documentation of adequate prior TB therapy, two had negative IGRA results, and only two completed LTBI treatment in the U.S. Conversely, no active TB developed among immigrants who were both IGRA-negative and had a normal immigration CXR. These findings suggest that high-risk immigrants with abnormal CXRs consistent with prior TB should be considered for preventive chemotherapy regardless of IGRA result.

As expected, active TB incidence was highest among 13 % of participants who were IGRA-positive at enrollment but received inadequate or no prior TB treatment. Our findings suggest that IGRA-positive immigrants are at high risk for progression to active TB disease, especially if they have not received adequate preventive chemotherapy, and efforts should be made to treat this group. The remaining 87 % of IGRA-positive participants did receive TB treatment and although the incidence rate for progression to active TB was more than 4 times lower than in those who had not received adequate therapy, the rate in treated individuals remained unacceptably high. It is unclear why preventive TB chemotherapy had only a modest impact on progression to active TB in our study. Preventive chemotherapy for LTBI was not typically delivered as directly observed therapy, so adherence was not guaranteed. Also, since treatment was not randomized, participants who were more likely to progress to active TB disease (such as those with a positive IGRA and evidence of prior TB by CXR) may have preferentially received preventive chemotherapy (confounding by indication).

More than 6 % of our initial cohort were diagnosed with prevalent active TB as a result of the enrollment evaluation. All but one of the prevalent cases had an abnormal immigration CXR consistent with imported active pulmonary TB. This high prevalence is consistent with other studies of recent immigrants [13, 29, 32], and highlights the importance of a thorough and timely TB evaluation of high-risk immigrants upon U.S. entry. In response to high numbers of imported active TB among newly arrived immigrants, the CDC has recently modified its pre-immigration screening to include sputum cultures instead of smears alone for TB suspects. This intensified overseas screening has resulted in a decrease in prevalent active TB among recent immigrants [29, 33, 34].

Our study had several limitations. We identified only seven cases of incident active TB despite following a large cohort for more than 7,000 person-years. This small number of outcomes led to imprecise incidence rate estimates and difficulty in determining the effects of multiple predictors such as IGRA status, LTBI treatment, and CXR abnormalities on active TB incidence rates. We were careful only to include true incident cases, excluding prevalent cases diagnosed as a result of enrollment screening and only including microbiologically or pathologically confirmed cases of active TB disease in the final analysis. Inclusion of clinically diagnosed cases would have increased incidence rates, but would have also increased the likelihood of misclassification of outcome. Case detection was limited by a passive follow-up and case finding approach. We identified cases reported in California but may have missed cases among those participants that moved out of state, resulting in an underestimation of incidence rates. Additionally, we were unable to censor follow-up time for participants that moved out of state or for all participants that died during the follow-up period, which would result in an overestimation of person-years at risk. These limitations would have led to a conservative, non-informative bias whereby incidence rates in both IGRA-positive and IGRA-negative participants were equally underestimated, assuming that moving and death were not associated with IGRA status. Second, although our case registries were rich sources of demographic and clinical data, reliance on registry data limited our ability to comprehensively characterize patients. Additionally, without TB genotypes for the active cases we were unable to confirm *M. tuberculosis* origin. Finally, this is a single center’s experience and reflects the largely Asian immigrant population of San Francisco. As such our findings might not be generalizable to other high TB burden areas in the U.S. such as southern California and Texas which experience a higher proportion of immigrants from Central and South America.

## Conclusions

Our study found a high prevalence of active TB disease among newly arrived immigrants and high incidence rates of active TB disease throughout a median of 6.7 years follow-up. Risk of progression to active TB disease was higher in IGRA-positive participants compared with IGRA-negative participants, although these findings did not reach statistical significance. Risk was highest among IGRA-positive participants who had received inadequate or no prior treatment for TB and lowest among IGRA-negative participants who had received prior treatment for TB or who had a normal CXR. Our findings support the role of timely and thorough TB screening of high-risk newly arrived immigrants from TB-endemic countries, preventive chemotherapy for IGRA-positive foreign-born persons from TB-endemic countries regardless of U.S. entry date, and preventive chemotherapy for those with radiologic evidence of prior TB if prior treatment cannot be verified, even if IGRA-negative. A positive IGRA by itself had poor predictive value for the progression to active TB disease, and additional research is needed to identify biomarkers and develop clinical algorithms that can better predict progression to active TB disease among U.S. immigrants.

## Abbreviations

ATS: American thoracic society
BCG: Bacillus Calmette-Guerin
BUL: Bilateral upper lobes
CDC: Centers for disease control
CXR: Chest x-ray
HIV: Human immunodeficiency virus
IDSA: Infectious diseases society of America
IGRA: Interferon-gamma release assay
IQR: Interquartile range
IRR: Incidence rate ratio
LLL: Left lower lobe
LTBI: Latent tuberculosis infection
LUL: Left upper lobe
RUL: Right upper lobe
SD: Standard deviation
SFDPH: San Francisco department of public health
TB: Tuberculosis
TST: Tuberculin skin test
